# Methazolamide Associated Stevens-Johnson Syndrome/Toxic Epidermal Necrolysis in a Female of Caucasian Descent

**DOI:** 10.7759/cureus.21864

**Published:** 2022-02-03

**Authors:** Amarah Baluch, Moeed R Chohan, Katerina Warda, Rishab Sippy, Jasmine Sandhu

**Affiliations:** 1 Internal Medicine, State University of New York Upstate Medical University, Syracuse, USA

**Keywords:** cutaneous adverse drug reaction, hla, toxic epidermal necrolysis, stevens johnson, methazolamide

## Abstract

Stevens-Johnson syndrome (SJS) is a potentially life-threatening cutaneous disorder that is characterized by skin erosions. It lies on a spectrum of varying severity with toxic epidermal necrolysis (TEN) being the most severe form. An overlap of the syndromes is known as SJS/TEN. These disorders are most often caused by a drug reaction, with anti-epileptic drugs and sulfonamide drugs as the common offending agents. Rarely, the syndrome can be due to a reaction to carbonic anhydrase inhibitors such as methazolamide. When present in association with methazolamide, the syndrome has only been known to occur in patients of Asian descent with human leukocyte antigen (HLA) mutations. We present a case of methazolamide-associated Stevens-Johnson syndrome in a patient of Caucasian descent.

## Introduction

Stevens-Johnson syndrome (SJS) is rare with an annual incidence in one to six per million people. As for toxic epidermal necrolysis (TEN), it has an incidence of 0.4 to 1.2 per million people [1]. 

Among the drugs that cause SJS, methazolamide has been reported to be the culprit drug approximately 13 times as of 2019 [2]. The cases of methazolamide-associated SJS have only been documented to occur in patients of Asian descent; its occurrence in this demographic is thought to be due to genetic differences [2]. We present a case of methazolamide-associated Stevens-Johnson syndrome/toxic epidermal necrolysis in a patient of Caucasian descent.

## Case presentation

An 85-year-old Caucasian female with a past medical history significant only for hypertension and glaucoma presented to the tertiary care center for a rash.

The patient had reported that three weeks prior to presentation she had been started on oral methazolamide by her ophthalmologist for glaucoma. Ten days into her medication course she developed a rash that, she reported, had initially started on her hands as small macules. At this point in time, she had stopped taking her methazolamide and had immediately presented to an urgent care center where she was advised to take Benadryl. Over the following two to three days, her rash progressively started to involve more areas of her body. She reported involvement of her entire trunk, her arms bilaterally, as well as her thighs. It became increasingly painful as well and she started to develop a subjective fever at home. She noted that in some areas, her rash had become bullous and also reported blisters on her hands, as well as on the soles of her feet. She felt there was some discomfort and involvement of her eyes as well. At this point in time, she presented to our facility. 

**Figure 1:**
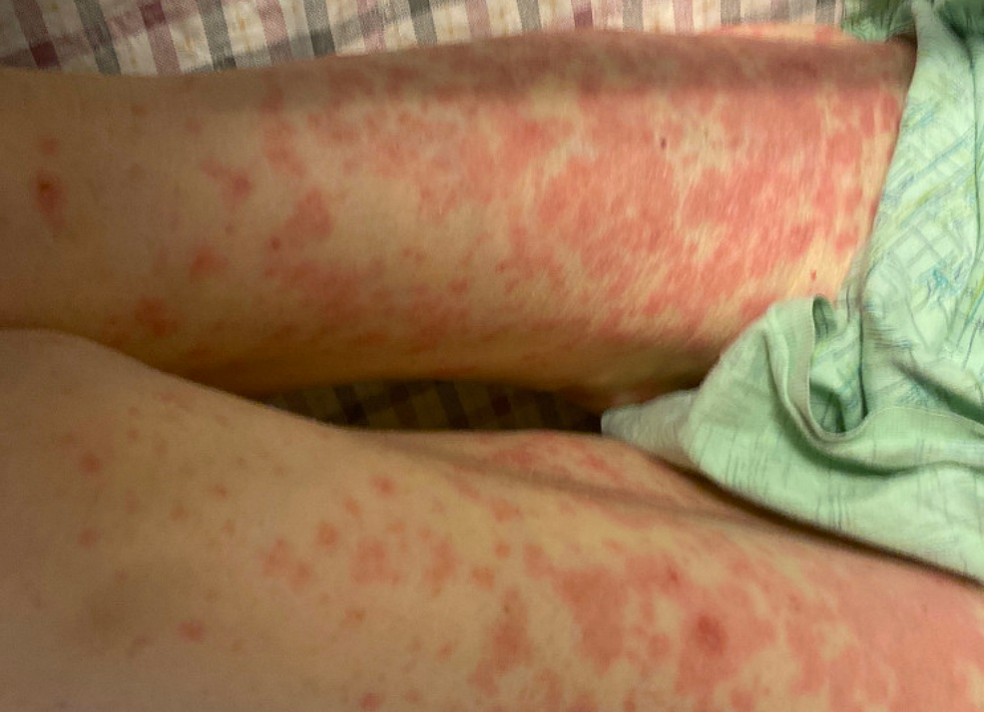
Rash involving the thighs bilaterally

Upon presentation at our hospital, she was hemodynamically stable. On physical exam, she was noted to have dry mucous membranes as well as erythema of her conjunctiva bilaterally. She was found to have a significant rash consisting of erythematous macules which were convalescing. It was noted that her rash involved her arms bilaterally, her entire abdomen, as well as her thighs as seen in Figure 1.

**Figure 2:**
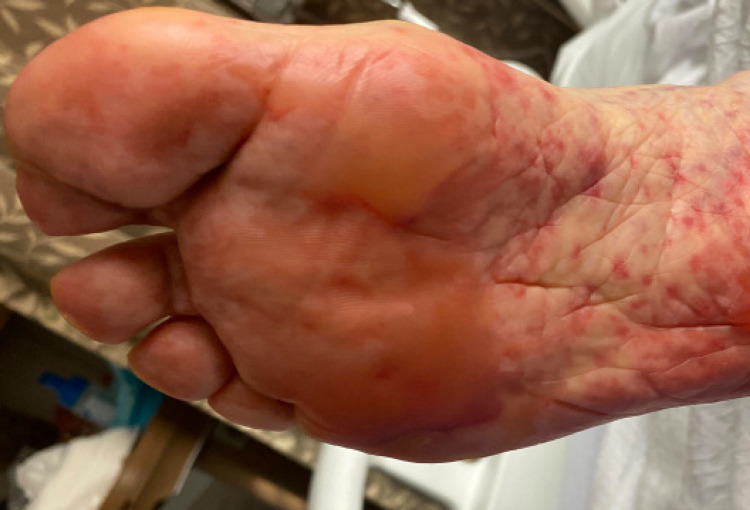
Involvement of the right foot with blistering

**Figure 3:**
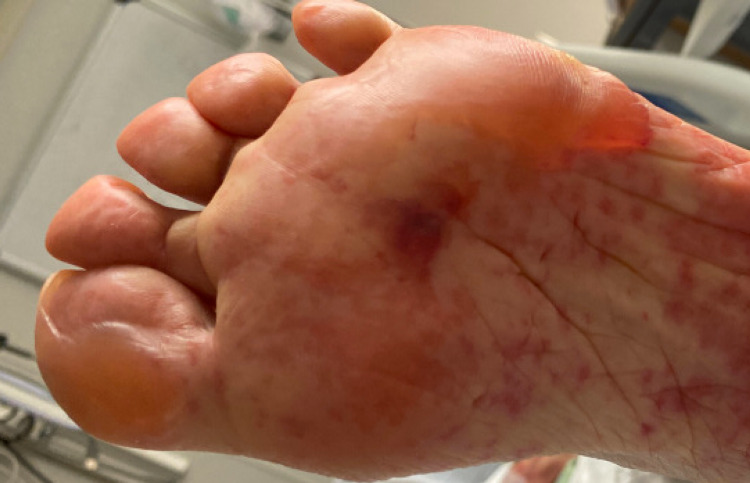
Blistering and sloughing of the left foot

**Figure 4:**
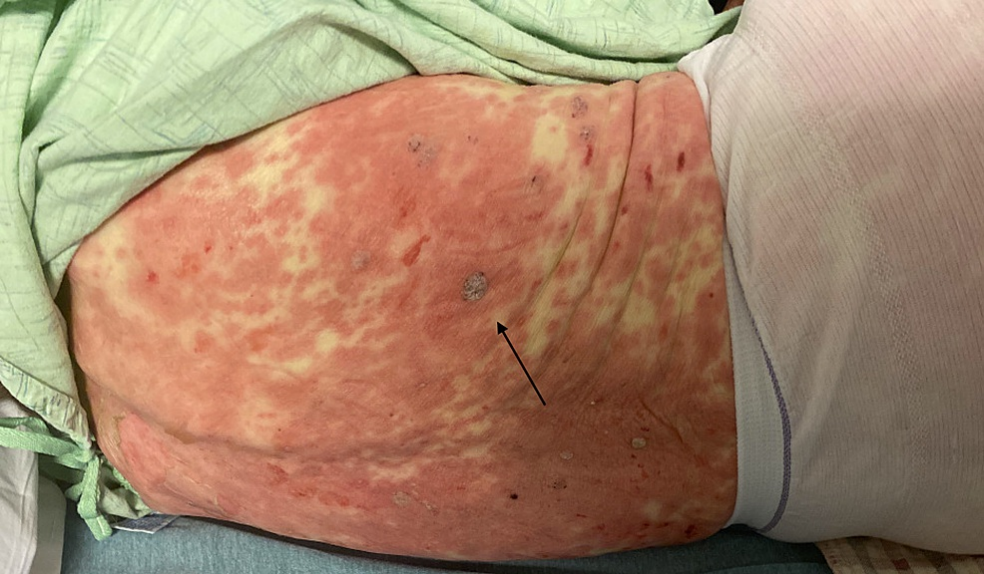
Involvement of the trunk with necrotic points (arrow)

She had a desquamating rash on her hands bilaterally. She was also noted to have sloughing on the soles of her feet with blistering as seen in Figures 2, 3, and sloughing of her lips. She had diffuse skin tenderness and some necrotic points on her trunk (Figure 4). She did not have any urogenital involvement in her rash. It was noted that approximately 20% to 30% of her total body surface area was involved with this rash. 

She was admitted for a presumed diagnosis of Stevens-Johnson syndrome. She was evaluated by the burn service due to concerns for Stevens-Johnson syndrome who suggested supportive treatment. She was also seen by ophthalmology due to ocular involvement for the same and they recommended moxifloxacin eye drops, cyclosporine eye drops and latanoprost eye drops. While admitted she underwent a punch biopsy of the skin and the results were consistent with interface dermatitis with necrotic epidermis, most consistent with erythema multiforme. These findings were confirmatory for Stevens-Johnson syndrome or toxic epidermal necrolysis. Given the percentage of surface area involvement, she was diagnosed with SJS/TEN. She was evaluated by the dermatology service who suggested she start steroids. She was started on IV prednisolone and was then transitioned to oral prednisone following which, the rash subsequently resolved. 

## Discussion

Stevens-Johnson syndrome is a rare, potentially life-threatening cutaneous disorder characterized by skin blistering and mucosal erosions. A more severe presentation of SJS is known as toxic epidermal necrolysis (TEN) and both syndromes lie on a spectrum where they are defined by varying degrees of severity and mortality. Stevens-Johnson syndrome is the more limited form and is defined by the involvement of less than 10% of total body surface area. Toxic epidermal necrolysis is the most severe version of this syndrome and is defined by greater than 30% of total body surface area involvement. An overlap of the two is known as SJS/TEN; in this form, there is the involvement of 10% to 30% of body surface area. Such was the case in our patient. All pathologies lying on the SJS/TEN spectrum can occur as a primary phenomenon, as a manifestation of a systemic pathology such as infection or malignant disease, and most commonly, as a reaction to drugs [3]. 

The drugs which are most often associated with SJS/TEN are allopurinol, anti-epileptic drugs such as phenobarbital, phenytoin, lamotrigine, carbamazepine, sulfonamide drugs, and oxicam non-steroidal anti-inflammatory drugs (NSAIDS) [4]. Carbonic anhydrase inhibitors such as methazolamide are also known to rarely be implicated in the syndrome. The findings of the Korean retrospective study conducted by Yang et al. to identify the culprit drugs found that most of the cases associated with methazolamide occurred between 13 and 44 days after starting the drug [5]. In the patient described above, her symptoms started approximately 10 days after the initiation of methazolamide. Interestingly, this study also found that carbonic anhydrase inhibitors were among the most common causes of SJS/TEN in South Korea while such is not the case in the United States. This difference is thought to be due to genetic differences [4,5]. 

Literature review suggests that when associated with methazolamide, the syndrome has been observed to only occur in patients of Asian descent in association with human leukocyte antigen B27 (HLAB2) [4,5]. Our patient was Caucasian with no Asian ethnic background known to the patient. This case is a rare documented case of methazolamide-associated SJS/TEN identified in a Caucasian patient. 

The clinical presentation of SJS is defined by a prodromal phase characterized by non-specific symptoms such as those of an upper respiratory tract infection. Patients may also have malaise and fever for up to two weeks. This prodrome is then followed by a rapid onset of a mucocutaneous eruption which is best described as macules with necrotic centers and blistering such as those seen in our patient in Figure 4. This eruption is seen in 90% of affected individuals. As the disease progresses, the lesions may become confluent [5]. The lesions often have a positive Nikolsky sign [6]. With further progression, the skin then begins to slough. Skin lesions are extremely painful and are not only limited to the superficial skin; there may be involvement of any of the mucous membranes, including the conjunctiva, oral cavity, nasal cavity and respiratory tract. There may also be genitourinary involvement.

There are no universally accepted criteria for the diagnosis of SJS/TEN. There are, however, several findings that can be used to help diagnose this syndrome. It is appropriate to consider SJS/TEN in a patient in whom there is a suggestive history of drug exposure or febrile illness where drug exposure precedes the onset of symptoms by one to four weeks, a prodrome of illness, a painful and rapidly progressive rash with erythematous macules and a positive Nikolsky sign [7]. There may also be necrosis and sloughing of the epidermis as seen in our patient, as well as oral, ocular or genital involvement. It is also necessary to assess drug causality. Another tool that may help with diagnosing is the algorithm of drug causality for epidermal necrolysis (ALDEN) criteria. The ALDEN criteria is an algorithm to assess for drug causality and potentially offending drugs are assigned a score from -11 to 10. These scores are assigned based on six parameters which then helps categorize the score as very probable, probable, possible, unlikely, and very unlikely [8]. The severity-of-illness score for toxic epidermal necrolysis (SCORTEN) score is another scoring system that may be used to assess the prognosis of patients. Neither of these scores is diagnostic. A skin biopsy will ultimately help confirm the diagnosis and exclude other SJS/TEN mimics [8]. Emerging tests include granulysin and high mobility group protein B1 levels but these have only been studied in small studies [7].

Some differentials to consider include erythema multiforme and drug eruptions [8]. Erythema multiforme will typically have less than 10% involvement of body surface area and is associated with the herpes simplex virus but rarely occurs with drugs. Biopsy findings may be the same. Drug eruptions tend to lack mucosal involvement and skin pain. In the patient described above, there was a greater than 10% body surface area involvement with mucosal involvement and significant pain, effectively ruling out these other differentials. 

Management principles consist first of discontinuing the offending agent and transferring to an intensive care unit or burn unit for supportive care [6]. The mortality rate may be reduced if patients are transferred to a burn unit given their ability to provide supportive care, which includes repair of the skin barrier, supportive fluids, and ocular care. Management will differ based on the organ system involved. It is also necessary for close monitoring for the prevention and treatment of superimposed infection. For integumentary involvement, it is advised to cover the skin with antibiotic-impregnated dressing. If there is ocular involvement then prompt ophthalmology consult is indicated with lubricating and topical antibiotic eye drops. There may also be pulmonary involvement which may require supplemental oxygen [8]. 

Outside of supportive treatment, there is no current established guideline regarding the use of systemic therapies [8]. There currently remains no adequate data regarding the use of systemic corticosteroids, immunosuppressants, intravenous immune globulin, cyclosporine or plasmapheresis. This is because these therapies have not been adequately studied in randomized controlled trials [8]. Systemic steroid use does remain controversial because early observational studies identified more complications and higher mortality in those patients treated with corticosteroids. However, other studies have shown that moderate to high dose systemic corticosteroids may not harm and may be beneficial if given early in the disease course [8]. The data remains controversial and contradictory, with some studies suggesting a potential benefit of systemic steroids but without any clear guidelines regarding treatment dosing, timing, or duration. There is also little evidence to support the use of intravenous immune globulin (IVIG) in SJS/TEN. Some studies have identified that the use of cyclosporine may slow the progression of SJS/TEN [8]. 

The mortality rate remains relatively elevated; in patients with SJS and TEN, overall mortality is estimated to be approximately 25%, with about 10% for SJS to more than 30% for TEN [8]. There is also a mortality risk due to SJS related complications from the syndrome such as sepsis, acute respiratory distress syndrome, and multi-organ failure. The most significant risk factor for mortality is disease severity which is defined by the extent of cutaneous involvement. There are also long term sequelae including cutaneous, mucosal, ocular, and pulmonary complications. To avoid recurrence, patients who develop SJS/TEN from medication exposure must understand to avoid the offending agent. 

This case highlights the importance of considering carbonic anhydrase inhibitors as a causative agent for SJS/TEN disorders. Certain populations are susceptible to certain agents based on underlying genetic differences. Patients who develop SJS/TEN from a certain class of drugs should be advised to avoid other agents of the same class. In fact, given that human leukocyte antigen groups are associated with some drug-induced SJS/TEN syndromes, family members of patients who develop the syndrome should also be advised not to use the same medication [8]. 

## Conclusions

Stevens-Johnson syndrome/toxic epidermal necrolysis is a rare, mucocutaneous disorder most often caused by a reaction to certain drugs. Within the drugs implicated, it is very rarely associated with methazolamide. The cases documented of methazolamide-associated SJS-TEN have only been reported in patients of Asian descent. Given the potential fatality of this disorder as well as no clear established guidelines on treatment, it is important to consider methazolamide as the culprit drug in patients of any ethnicity. It is also important to revisit prescribing habits of this drug especially given that certain populations are more susceptible to SJS/TEN when exposed to methazolamide.
